# Transient suppression of Wnt signaling in poor-quality buffalo oocytes improves their developmental competence

**DOI:** 10.3389/fvets.2023.1324647

**Published:** 2024-01-11

**Authors:** Kriti Ahuja, Vipul Batra, Rakesh Kumar, Tirtha Kumar Datta

**Affiliations:** ^1^Animal Genomics Lab, Animal Biotechnology Centre, ICAR-National Dairy Research Institute, Karnal, India; ^2^Lifespan and Population Health, School of Medicine, University of Nottingham, Nottingham, United Kingdom; ^3^ICAR-Central Institute for Research on Buffaloes, Hisar, India

**Keywords:** Brilliant Cresyl Blue (BCB) screening, oocyte quality and competence, Wnt pathway, *in vitro* embryo production (IVEP), buffalo

## Abstract

**Introduction:**

One of the most evolutionary conserved communication systems, the Wnt signaling pathway is a major gene regulatory pathway that affects the developmental competence of oocytes and regulates most embryonic developmental processes. The present study was undertaken to modulate the canonical Wnt (Wingless/integration) signaling pathway in the poor-quality (colorless cytoplasm after Brilliant Cresyl Blue staining, BCB-) buffalo cumulus-oocyte complexes (COCs) to improve their *in vitro* maturation (IVM) and embryo production (IVEP) rates.

**Methods:**

The expression of key Wnt pathway genes was initially assessed in the good (blue cytoplasm after Brilliant Cresyl Blue staining, BCB+) and poor quality (BCB-) buffalo COCs to establish a differential activity of the Wnt pathway. The BCB- COCs were supplemented with the Wnt pathway inhibitor, Dickkopf-related protein 1 (DKK1) and later subjected to IVM and IVEP along with the BCB+ and BCB- controls. The cumulus expansion index (CEI), rate of nuclear maturation (mean percentage of oocytes in the MII stage) and embryo production, and the expression of developmentally important genes were evaluated to assess the effect of Wnt pathway inhibition on the development competence of these poor-quality oocytes.

**Results:**

The Wnt pathway genes exhibited a significantly higher expression (*p* < 0.05) in the poor-quality BCB- oocytes compared to the good-quality BCB+ oocytes during the early maturation stages. The supplementation of BCB- COCs with 100 ng/mL DKK1 effectively inhibited the expression of the key mediators of the Wnt pathway (β-catenin and dishevelled homolog 1, DVL1). DKK1 supplemented BCB- COCs exhibited significantly improved cytoplasmic and nuclear maturation indices, development rates and significantly elevated expression (*p* < 0.05) of genes implicated in germinal vesicle breakdown (GVBD) and embryonic genome activation (EGA) vis-à-vis BCB- control COCs.

**Conclusion:**

These data indicate that inhibition of the Wnt pathway during the initial course of oocyte maturation can improve the development competence of poor-quality buffalo oocytes.

## 1 Introduction

An irreplaceable milk producer in tropical countries, Buffalo suffers from several reproductive limitations, e.g., silent estrous expression, low conception rate, seasonal fluctuations in fertility, long calving intervals and a low number of primordial follicles (1–3). Adept implementation and use of assisted reproductive technologies (ARTs) have been suggested as measures to overcome these physiological limitations and for the fast propagation of superior germplasm (4–7). Despite the availability of highly optimized culture conditions and techniques for the stringent quality selection of oocytes, the *in vitro* embryo production (IVEP) technology in buffalo has not been very successful (8, 9). Such optimized methodologies that are otherwise successful in cattle often result in only a meager percentage of buffalo oocytes reaching the blastocyst stage (10, 11). The development of *in vitro* fertilized buffalo embryos depends upon numerous extrinsic and instric factors viz. ovarian morphology, season, donor, culture conditions, hormones, and oocyte quality among others (7, 12–14). Besides, the low efficiency of multiple-ovulation and embryo transfer (MOET) often attributed to low fertility and poor response to superovulation further decrease the success rate of buffalo IVEP (4, 9). The identification and selection of good-quality oocytes (high competence) is a crucial step for ensuring their complete maturation and subsequent embryonic development, which is critical for successful IVEP (15–17). The Brilliant Cresyl Blue (BCB) staining framework, which assesses the physiological status of oocytes has been used as a successful methodology for selecting competent oocytes (18–20). This technique relies on the activity of glucose-6-phosphate dehydrogenase (G6PD) in the growing oocytes. A low G6PD activity results in blue coloration of the cytoplasm (BCB+ oocytes) indicating completion of oocyte maturation/growth (21, 22). Contrarily, the BCB- oocytes reduce the BCB stain and appear colorless post-staining. Such oocytes lie far behind the BCB+ oocytes in terms of their developmental potential (23). One of the greatest challenges that still exists in the field of reproduction and developmental biology is to understand what constitutes oocyte developmental competence.

The Wnt pathway is considered one of the major gene regulatory pathways that affect the developmental competence of oocytes and is considered crucial for several early developmental processes, e.g., *in-vitro* blastocyst development (24–27). One of the most evolutionary conserved communication systems, the Wnt pathway regulates most embryonic developmental processes, e.g., dorsoventral and anteroposterior body axes formation, cell fate determination and organogenesis (28–31). A wide range of effectors (agonists/antagonists) regulates the Wnt pathway and these effectors can act either intracellularly or extracellularly. At the intracellular level, they modify the components of the signaling machinery whereas, at the extracellular level, they modulate the ligand-receptor interactions (32, 33). These antagonists and agonists are of immense importance as they help in the intricate modulation of the Wnt signaling and thus help in the activation or inhibition of Wnt-regulated developmental processes (34). One such antagonist, Dickkopf-Related Protein 1 (DKK-1) is a secreted inhibitor of the Wnt pathway that belongs to the Dickkopf family of evolutionarily conserved glycoproteins (35, 36). DKK1 inhibits the Wnt/β-catenin pathway by binding to the essential co-receptors for Wnt signaling, i.e., low-density lipoprotein receptor-related protein 5 (LRP5) and LRP6 which results in decreased β-catenin protein stability (37). The effect of DKK1 on oocyte maturation and function, embryo development, hatching of blastocysts and their implantation has been studied in detail (38–42). For example, the supplementation of Dickkopf-Related Protein 1 to the maturation medium of *in vitro* matured oocytes (with increased Wnt gene expression) improved their maturation parameters and the number of nuclei in the resulting blastocyst stage embryos (43). The abovementioned studies revealed that the modulation of the Wnt signaling pathway is critical for IVEP since the dysregulated activity in the Wnt pathway can perturb the embryonic development process. Therefore, we hypothesized that a differential level of Wnt signaling activity exists in the buffalo oocytes of high (BCB+) and low (BCB-) quality and a transient induction/repression of this pathway should improve the development competence of poor-quality oocytes.

Thus, the primary objective of the current study was to investigate whether inhibiting/activating this pathway at a pre-determined course of oocyte maturation can help the buffalo oocytes of poor quality (BCB-) gain enhanced development competence.

## 2 Methods

All chemicals, media and reagents were procured from Sigma Aldrich Chemical Co. Ltd., (United States) unless stated otherwise and plastic ware was obtained from Nunc (Thermo Fisher Scientific, USA). Fetal bovine serum (FBS) was obtained from Hyclone, Canada.

### 2.1 COCs (cumulus-oocyte complexes) collection, BCB screening, and *in vitro* maturation

The collection of COCs and BCB screening were done as described previously for buffalo (44) with minor modifications. Briefly, the buffalo ovaries were collected from a local abattoir (Delhi) and were transported within 4 h to the laboratory in physiological saline (0.9%, w/v NaCl, 38°C) containing Streptomycin–penicillin (50 mg/L). The cumulus-oocyte complexes (COCs) were aspirated from the antral follicles (4–8 mm in diameter) using an 18G needle attached to a 10 mL syringe containing 1 mL Hepes-buffered hamster embryo culture (HH) medium. The quality of COCs was observed under a stereo-zoom microscope (20X magnification) and only the grade A COCs (> 4 layers of cumulus cells and clear, homogenous cytoplasm) were considered for downstream experiments.

The selected grade-A COCs were washed thrice in HH medium, subsequently exposed to 26 μM BCB solution and incubated for 90 min at 38.5°C in a CO₂ incubator. The excess stain was removed by washing once in mDPBS and the COCs were observed under a stereo zoom microscope. COCs were classified based on cytoplasm coloration as either BCB+ (Blue cytoplasm) or BCB- (Colorless cytoplasm). Later, both the BCB+ and BCB- COCs were kept in IVM medium (HEPES buffered TCM-199 supplemented with 10% FBS, 0.14 mg/mL Sodium Pyruvate, 0.1 mg/mL Glutamine, 5 mg/mL Streptomycin, along with 0.5 mg/mL FSH, 0.5 mg/mL LH, 1 μg/mL Estradiol 17-β, 0.1 mg/mL EGF, and 32 mg/mL Cysteamine) for washing. The washed COCs were then placed in 100 μL maturation drops (*n* = 15 COCs/drop) prepared in 35 mm culture dishes and were then overlaid with 3 mL of mineral oil. The COCs were incubated at 38.5°C in a CO₂ incubator with 98% humidity and 5% CO₂ for 24 h.

### 2.2 Expression analysis of Wnt pathway genes in BCB+ and BCB- oocytes

For RNA isolation, the denuded BCB+ and BCB- oocytes (n = 10 each) were collected from maturation drops at different hours of maturation, i.e., 0, 8, 16, and 24 h. The oocytes were denuded by brief vortexing followed by repeated washing in PBS (Phosphate Buffer Saline). The 10 oocytes were pooled together for RNA extraction and the experiment was carried out in three biological replicates. The total RNA was isolated using the RNAqueous Micro Kit (Ambion, United States) according to the manufacturer’s instructions and quantified using a NanoDrop ND-1000 UV–Vis spectrophotometer (NanoDrop Technologies Inc., Wilmington, DE, United States). Only the samples with A260/280 and A260/230 close to 2.0 were considered for the gene expression experiments. All the RNA samples were treated with RNase-free DNase I (Ambion, United States) and a fixed amount of RNA (100 μg) was reverse transcribed into cDNA using the RevertAid, H Minus first-strand cDNA synthesis kit (Thermo Scientific, USA) following the manufacturer’s instructions. A reverse transcriptase negative control was prepared to assess the residual genomic DNA contamination in the RNA sample. The cDNA samples were diluted three times (final volume-60 μL) with nuclease-free water and stored at −20°C until further use.

To study the differential activity of the Wnt pathway in BCB+ and BCB- oocytes, the gene expression of the key molecules of the Wnt pathway viz. β-catenin, DVL1, FZD4, WNT3A, and WNT7A were quantified. The intron-spanning primers for the Wnt pathway genes and the reference gene (RPS18) were designed using the Primer 3 software using the sequences obtained from the bovine genome database in GenBank and EMBL (Supplementary Table 1). The self-annealing sites, mismatches and secondary structures in the designed primers were checked using Oligo Calc (45). The specificity of primers was checked using the BLAST alignment tool (46) and *in silico* PCR (47) was run for each set of primers before commercial synthesis (Sigma-Aldrich, United States). To ensure the quality of RT-qPCR data, MIQE guidelines (48) were followed at every step, wherever possible. The relative quantification of the Wnt pathway genes was done on a Roche LightCycler® 96 platform (Roche Diagnostics, Mannheim, Germany) using the Maxima SYBR Green qPCR master mix (Fermentas, United States) in a 10 μL reaction mix. The reactions were performed in duplicate for each sample with the following thermal profile: denaturation at 95°C for 10 min, then 40 cycles consisting of denaturation at 95°C for 15 s, annealing at optimized temperatures for 15 s and extension at 72°C for 20 s. Upon completion of amplification, a melt curve analysis was performed to ensure a specific, unique product formation and to ascertain minimal primer-dimer formation. A no-template control (NTC) was run in each plate to confirm the absence of nucleic acid contamination. The mean sample Cq (Cycle of quantification) values for different transcripts were calculated for the duplicate samples and the relative expression ratio of the Wnt pathway gene expression was calculated using the ΔΔCt (Cycle threshold) method (49).

### 2.3 Validation of the differential gene expression between the BCB+ and BCB- oocytes

To validate the differential gene expression between the BCB+ and BCB- oocytes at the protein level, we measured the abundance of β-catenin, a key component of the Wnt pathway (50) at 0, 8, 16, and 24 h of maturation. Briefly, the cumulus cells were separated from the BCB+ and BCB- COCs by vortexing followed by washing in PBS (phosphate-buffered saline). The oocytes were then fixed in freshly prepared 4% (w/v) paraformaldehyde in PBS for 30 min at RT (Room Temperature) and subsequently permeabilized in 0.5% Triton X-100 (in PBS) for 10 min at RT. Thereafter, the oocytes were washed with 0.05% Tween-20 (PBST) and blocked with 1% BSA (Bovine Serum Albumin) for 1 h and subsequently transferred to primary antibody solution (Abcam, ab6302, Rabbit polyclonal to β-catenin, 1:100 in PBST) and incubated at 4°C overnight. The next day, the oocytes were washed 10 times (5 min) in PBST containing 1% BSA and transferred to a FITC conjugated secondary antibody (Abcam, ab97050, 1:500) solution for 30 min and mounted on a glass slide using Prolong Gold Antifade Reagent and observed under a blue filter (Fluorescence filter characteristics: excitation wavelength 470-490 nm, emission wavelength 510 nm) of a fluorescent microscope (Olympus BX-51, fitted with a digital CCD, DP71 camera) using the Olympus MICRO DP71 software ver.03.03. The fluorescent intensity was measured and quantified for a minimum of 50 oocytes in at least 10 fields covering the entire slide (*n* = five biological replicates) using the Image J software. A “no-primary antibody” control was used to ensure signal from the detection of the antigen (β-catenin) by the primary antibody. The entire periphery of the oocyte was selected as the region of interest (ROI) and was considered for the quantification of the fluorescence signal. The fluorescence intensities were quantified at random from the micrographs that were captured using similar acquisition settings, e.g., the time of exposure, magnification and acquisition formats. The integrated density (IntDen) value, which represents the product of “Area”, and “Mean Gray Value” for the chosen ROIs in a non-calibrated image, was calculated for each cell. Thereafter, the IntDen values were normalized (vis-a-vis the immediate background) and considered the final mean fluorescent intensity (MFI) values.

### 2.4 Dosage determination of Wnt pathway inhibitor, DKK1

To inhibit the over activity of Wnt signaling in BCB- oocytes, Wnt-inhibitor, DKK-1 (50 ng/L, 100 ng/mL, and 200 ng/mL) was added to the IVM medium for the initial 8 h. After exposure of 8 h the BCB- oocytes supplemented with inhibitors were taken out from the IVM medium, washed thrice in HH media and kept in the IVM medium (without inhibitors). To select the optimum dose of the inhibitor, the cumulus expansion index (CEI) and embryo production rates were assessed.

### 2.5 Cumulus expansion and *in vitro* embryo production rates after DKK1 supplementation

After the supplementation of DKK1 for 8 h, the BCB- COCs were evaluated for their level of cumulus expansion after 24 h of IVM. A scale of 0–3 was used to measure the cumulus expansion. The COCs which had the maximum degree of cumulus expansion were assigned scale 3, COCs with no cumulus expansion were given scale 0 and those with intermediate expansion were given scale 1 (only outer layers expand) or 2 (more than 50% of layers expand).

To assess the *in vitro* embryo production rate upon DKK1 exposure, oocytes from distinct groups viz. BCB+, BCB-(Control), BCB-(+50 ng/mL DKK1), BCB-(+100 ng/mL DKK1), BCB-(+200 ng/mL DKK1) were processed for IVM separately. The *in vitro* fertilization (IVF) was carried out as per the standard protocol followed in our lab (51) in IVF droplets (100 μL) of Brackett and Oliphant (BO) medium supplemented with 1% BSA (fatty acid-free), 1.9 mg/mL Caffeine Sodium Benzoate, 0.14 mg/mL Sodium Pyruvate and 0.01 mg/mL Heparin. The matured oocytes were transferred into the drops after washing thrice in BO medium. The frozen buffalo semen was thawed simultaneously and processed for *in vitro* capacitation, as described previously (52). Fifty microliter of IVF media (BO) was replaced with 50 μL of the sperm suspension (1 × 10^6^ spermatozoa/mL) added to each fertilization drop with 15 COCs and then incubated at 38.5°C with 5% CO_2_ for 12 h. Thereafter, the presumptive zygotes were denuded by brief vortexing and washed five times in a modified Charles and Rosenkrans 2 amino acid (mCR2aa). After washing, the 15 presumptive zygotes were co-cultured with the monolayer of granulosa cells in 100 μL drops of IVC-I medium (mCR2aa supplemented with 8 mg/mL BSA, 180.156 mg/mL Glucose, 29.059 mg/mL Pyruvate, 146.14 Glutamine, 1 × MEM essential amino acid, 1x non-essential amino acid and 50 μg/mL Gentamycin). After 48 h post insemination (hpi) the zygotes were evaluated for evidence of cleavage. At 72 hpi, all the cleaved embryos were transferred to IVC-II medium (IVC-I with 10% FBS) and were maintained for 8 days post-insemination at 38.5°C with 5% CO_2_. The culture medium was regularly replaced every 48 h.

### 2.6 Inhibition of the Wnt pathway by DKK1 dosage during the initial stages of maturation

To ascribe the observed improvements in CEI and IVEP in BCB- oocytes upon DKK1 supplementation (100 ng/mL) to the inhibition of the Wnt signaling pathway, the expression of two key mediators of Wnt signaling viz. β catenin and DVL1 was evaluated at five different intervals, i.e., 0 h 2 h, 4 h, 6 h, 8 h of *in vitro* maturation in *n* = 10 oocytes/group (3 biological replicates). These time points were selected since the difference in the expression of Wnt genes in the BCB+ and BCB- oocytes was maximum during the initial 8 h of IVM.

### 2.7 Assessment of nuclear maturation of oocytes upon Wnt pathway inhibition

The nuclear maturation of oocytes at the end of IVM was ascertained by Hoechst staining, a simple and fast technique often used for chromatin evaluation. The cumulus cells were removed from the COCs by vortexing and subsequently washed thrice in PBS-PVP (1 mg/mL). The oocytes were then placed in Hoechst staining solution (10 μg/mL in PBS-PVP) for 15 min at 37°C and again washed (3X). The 12 washed oocytes were placed singly on a clean, grease-free microscopic glass slide having rails of wax along its two sides. The slides were observed under UV fluorescence (Fluorescence filter characteristics: excitation wavelength 355-375 nm, emission 420 nm) on an Olympus BX-51 microscope fitted with a DP-71 camera.

### 2.8 Effect of Wnt pathway inhibition on blastocyst health

The ICM/TE ratio, which is an indicator of the health of blastocysts, was determined using the differential staining of *in vitro*-produced blastocysts, as described by Kumar and co-authors (53). Briefly, the blastocysts were removed from the culture and washed with PBS-PVP before being placed in PI solution (100 μg/mL of Propidium iodide in 0.2% Triton X-100) for 20 s. Thereafter, the blastocysts were washed thrice in PBS-PVP (1 mg/mL) and placed in working Hoechst solution (1 μg/mL Hoechst 33342 prepared in 4% formaldehyde (40 mg/mL), in PBS-PVP) for 20 min. The blastocysts were placed on a clean, grease-free microscopic slide in a drop of glycerol and visualized under UV fluorescence and green filter using an Olympus BX-51 fluorescent microscope fitted with a DP-71 camera (Fluorescence filter characteristics: UV- excitation wavelength 355-375 nm, emission 420 nm and Green- excitation wavelength 520-550 nm, emission 580 nm). The two images were overlaid to form a merged image.

### 2.9 Relative quantification of developmentally important genes after Wnt inhibition

To analyze the effect of Wnt inhibition by DKK1 on oocyte competence, the expression pattern of various genes involved in oocyte maturation and embryonic development was studied. The dynamics of genes related to cumulus expansion (GDF9, HAS2, PTGS2) and GVBD (AKT, Cyclin B and CDC25B) was studied during 0, 2, 4, 6, and 8 h of IVM while the expression of genes related to EGA, i.e., Embryonic Genome Activation (eIF1A, U2AF, and PAP) was evaluated at 2 cell, 4 cell, 8 cell and 16-cell stages (Supplementary Table 1). Briefly, a pool of either 10 oocytes or 10 fertilized oocytes (at 2-cell, 4-cell, 8-cell, and 16-cell stages; 3 biological replicates) from the three sample groups viz. BCB+, BCB- (control), and BCB- supplemented with 100 ng/mL DKK1 were washed with chilled PBS and immediately processed for RNA isolation. The cDNA synthesis and RT-qPCR were carried out as described previously.

### 2.10 Statistical analysis

Statistical evaluation of the data was done using MS Excel and GraphPad Prism (GraphPad Software, San Diego, CA). The generated data was examined for normality of distribution and transformed if required using arc-sine transformation (Embryo development rate data). Analysis of variance (ANOVA) was used to determine the significance of experimental variables followed by Bonferroni Post-hoc tests to compare the significance of the effect of treatment between groups at 95% confidence intervals (*p* < 0.05). All the experiments were performed in at least three biological replicates and presented as Mean + SEM (Standard Error of Mean) (standard error of mean).

## 3 Results

### 3.1 Wnt pathway genes are differentially expressed in BCB+ and BCB- oocytes

**Figure 1:**
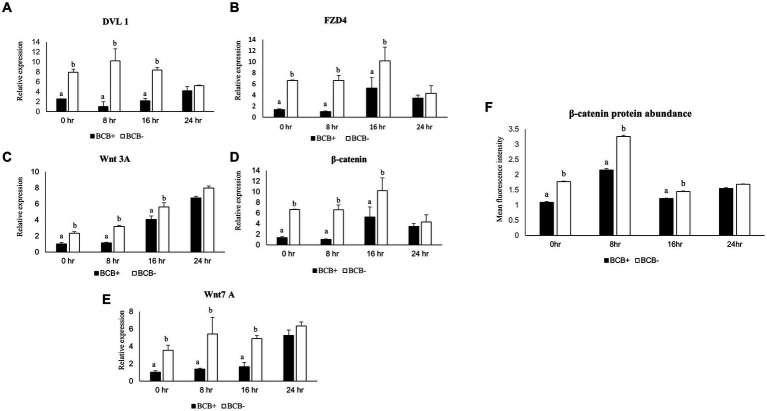
Differential expression of Wnt pathway genes in good and poor-quality buffalo oocytes. Relative expression profiles of Wnt pathway genes in BCB+ and BCB- oocytes during four different time points of maturation **(A–E)**. Mean fluorescence intensities obtained for β-catenin abundance by immunostaining in BCB+ and BCB- oocytes by using Image J **(F)**. Values are presented as Mean ± SEM. Bars with different superscripts indicate a significant difference (*p* < 0.05).

The expression pattern of a panel of five key members of the Wnt signaling pathway viz. β-catenin, Dvl1, FZD4, WNT3A, and WNT7A was examined in the BCB+ and BCB- oocytes at 0, 8, 16, and 24 h of IVM. All the genes considered exhibited a significantly higher expression in BCB- COCs compared to BCB+ COCs at all the time points, particularly during the initial hours of oocyte maturation (Figures 1A–E). The expression of β catenin and FZD4 was highest at 16 h post-IVM in BCB- oocytes whereas the expression of Dvl-1 and WNT3A peaked at 8 and 24 h, respectively. The differences in expression between the BCB+ and BCB- COCs were greatest at 8 h of IVM for all the Wnt pathway genes.

### 3.2 A higher abundance of β-catenin protein was observed in BCB- oocytes

β-catenin, a pivotal player in the Wnt pathway was found to be localized below the buffalo oocyte plasma membrane (Supplementary Figure 1), as reported previously in mice (54). The mean fluorescent intensity (MFI) for β-catenin was considerably higher (*p* < 0.05) in the BCB- oocytes vis-à-vis the BCB+ oocytes during the early course of maturation (Figure 1F). The maximum MFI was observed at 8 h post-IVM in BCB- COCs. Interestingly, the differences in abundance between the BCB+ and BCB- COCs were highest at 8 h post-IVM indicating a correlation between the gene expression and protein abundance. This established that the Wnt pathway is more active in BCB- oocytes than BCB+ oocytes, particularly during the initial 8 h of IVM. Therefore, we considered the first 8 h of maturation for manipulating the Wnt pathway in BCB- oocytes and carried out all further experiments at this time point.

### 3.3 Supplementation of DKK1 (100 ng/mL) improves CEI and development rates of BCB- oocytes

**Figure 2:**
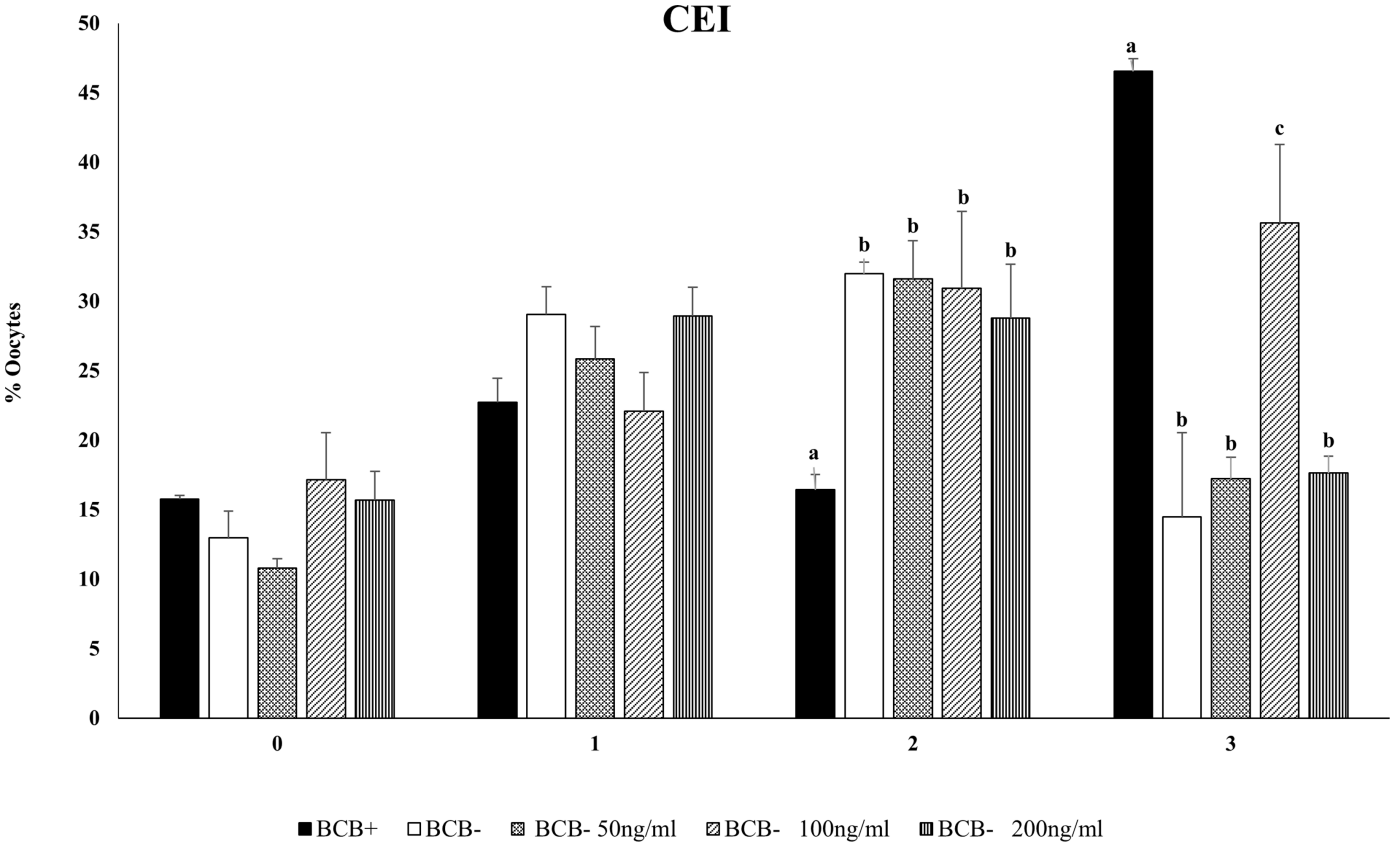
Cumulus expansion index (CEI)Mean percentage of oocytes observed with different cumulus expansion index in BCB+, BCB- (control), and BCB- oocytes supplemented with various concentrations of Wnt inhibitor, DKK1. Values are presented as Mean ± SEM. Bars with different superscripts indicate a significant difference (*p* < 0.05).

Cumulus expansion can be considered a morphological indicator of the oocytes’ fertilizing ability and is associated with higher developmental competence (55). To evaluate the effect of Wnt inhibition on cumulus expansion and *in vitro* embryo development, we considered three doses viz. 50, 100, and 200 ng/mL of Wnt inhibitor, DKK1 based on previous literature (43). The mean percentage of BCB+ oocytes with the maximum expansion, i.e., CEI-3 was significantly higher (*p* < 0.05) in the BCB+ oocytes compared with BCB- oocytes (Figure 2; Supplementary Figure 2). The supplementation of BCB- oocytes with 100 ng/mL of Wnt inhibitor assisted more oocytes in reaching the highest scale of cumulus expansion in buffalo oocytes.

The effect of Wnt inhibition on the development rate of buffalo oocytes was assessed by calculating the percentage of cleaved oocytes, those reaching 8-16cell, morula, and blastocyst stages of the embryo development. The inhibition of the Wnt pathway by DKK1 did not affect the initial stages of embryo development at any of the considered doses (Table 1). However, a significant difference (*p* < 0.05) in the mean percentage of fertilized oocytes reaching the morula and blastocyst stages was observed between BCB- oocytes supplemented with 100 ng/mL of Wnt inhibitor and the BCB- control.

**Table 1:** Developmental rates of BCB+, BCB-, and BCB- oocytes supplemented with various concentrations of Wnt inhibitor, DKK1.

Culture group	Total oocytes	% Cleaved	% 8–16 cell	% Morulae	% Blastocyst
BCB +	146	60.33 ± 0.941^a^	62.47 ± 1.92^a^	46.5067 ± 1.82^a^	23.94 ± 2.37^a^
BCB- (control)	131	29.63 ± 0.80^b^	36.11 ± 2.77^b^	30.55 ± 2.77^b^	9.722 ± 1.38^b^
BCB- + 50 ng/mL DKK1	125	31.85 ± 0.90^b^	39.01 ± 2.15^b^	36.63 ± 4.22^b^	12.08 ± 2.19^b^
BCB- + 100 ng/mL DKK1	120	32.15 ± 1.01^b^	43.21 ± 3.48^b^	43.39 ± 1.46^c^	16.73 ± 2.91^c^
BCB- + 200 ng/mL DKK1	141	31.13 ± 0.81^b^	34.06 ± 3.01^b^	31.64 ± 3.15^b^	13.65 ± 0.85^b^

The percentage of cleavage oocytes was calculated from the total number of oocytes while the percentage of 8–16 cell, morulae and blastocysts was calculated from the number of cleaved oocytes. Values are presented as Mean ± SEM. Bars with different superscripts indicate a significant difference (*p* < 0.05).

Based on the developmental data and cumulus expansion index we considered 100 ng/mL dose of DKK1 as the optimum dose for inhibition of Wnt and for carrying out our subsequent experiments.

### 3.4 DKK1 is an effective antagonist of the Wnt signaling pathway during the initial stages of IVM

**Figure 3:**
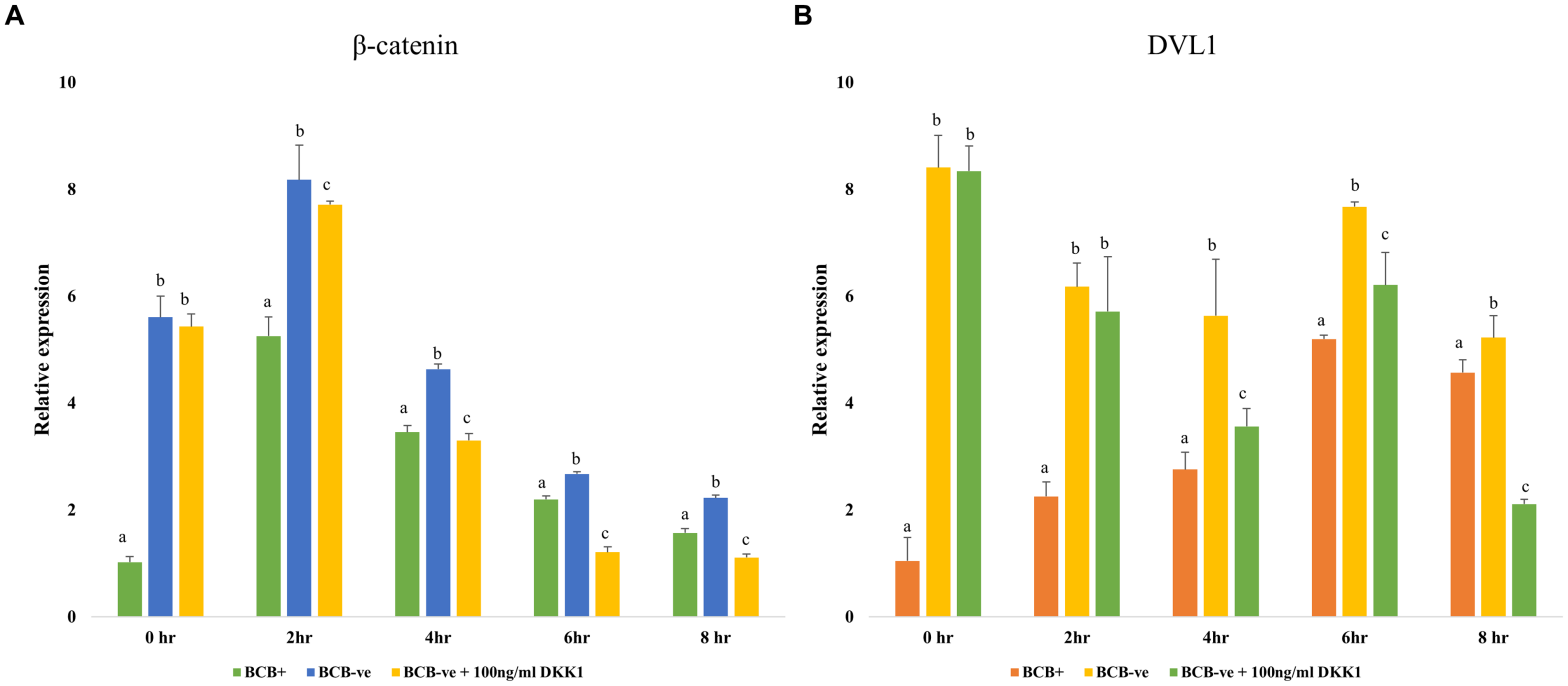
Wnt pathway inhibition Relative expression profiles of key Wnt pathway mediators β catenin **(A)** and DVL1 **(B)** in BCB+, BCB- (control), and BCB- oocytes supplemented with 100 ng/mL Wnt inhibitor, DKK1. Values are presented as Mean ± SEM. Bars with different superscripts indicate a significant difference (*p* < 0.05).

The expression of the key mediators of the Wnt signaling pathway viz. β catenin and DVL1 was observed to be significantly reduced (*p* < 0.05) in the BCB- oocytes supplemented with 100 ng/mL DKK1 compared to the BCB- (control) oocytes at 2, 4, 6, and 8 h of IVM indicating successful inhibition of Wnt pathway by DKK1 (Figure 3).

### 3.5 Inhibition of the Wnt signaling pathway increased the nuclear maturation rate of BCB- oocytes

A total of n = 12 oocytes/group were subject to Hoechst staining to evaluate the nuclear maturation rate of oocytes in the MII stage among the treatment and control groups considered. As expected, the BCB+ oocytes had a significantly higher percentage (*p* < 0.05) of oocytes in the MII stage than BCB- oocytes (Table 2). The rate of nuclear maturation increased significantly (*p* < 0.05) in the BCB- oocytes supplemented with 100 ng/mL DKK1 in comparison to the oocytes in the BCB- (control) group.

**Table 2:** Percentage of oocytes in MII stage in BCB+, BCB- (control) and BCB- oocytes supplemented with 100 ng/mL Wnt inhibitor, DKK1.

Group	Total oocytes	% Oocytes in MII stage
BCB+	60	61.667 ± 4.409^a^
BCB-	60	33.33 ± 3.333^b^
BCB- + 100 ng/mL DKK1	60	53.34 ± 1.667^c^

Values are presented as Mean ± SEM. Bars with different superscripts indicate a significant difference (*p* < 0.05).

### 3.6 DKK1 Supplementation does not promote cell proliferation in early embryos

**Figure 4:**
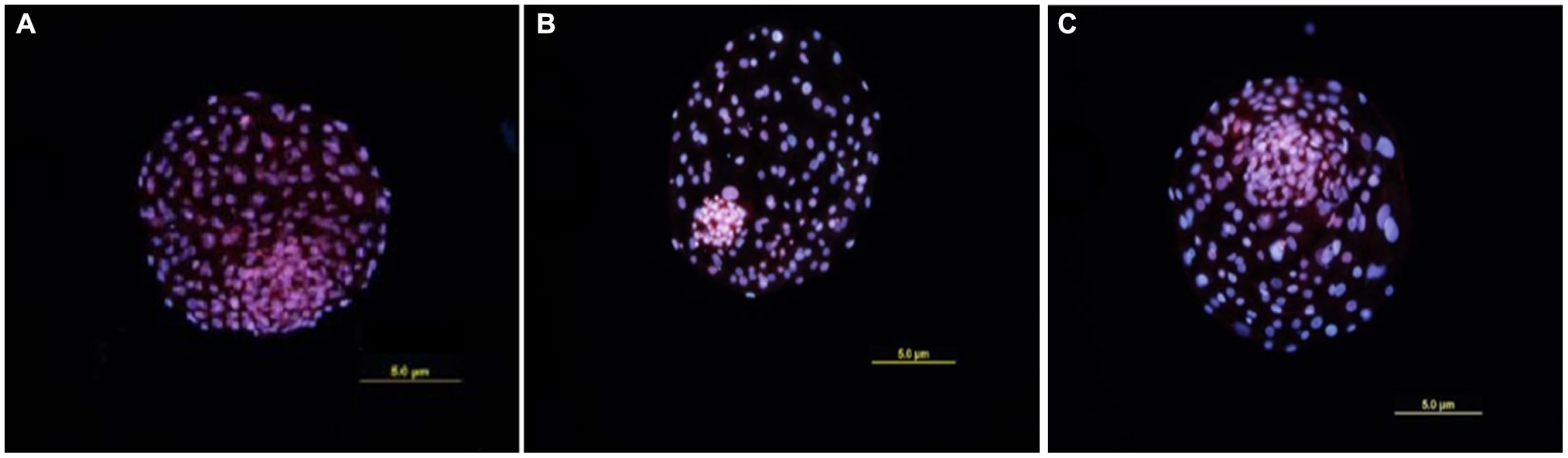
Differential Staining of blastocysts. Cells showing blue fluorescence are ICM cells while red fluorescent cells are TE cells. **(A)** Blastocyst produced by BCB+ oocytes. **(B)** Blastocyst produced by BCB- oocytes. **(C)** Blastocyst produced by BCB- oocytes supplemented with 100 ng/mL DKK1.

Five blastocysts per sample group were subject to differential staining for calculating the ICM/TE ratio. The ICM cells fluoresced blue (Hoechst) whereas the cells which showed reddish-pink fluorescence (Propidium iodide) were considered TE cells (Figure 4). The ICM/TE ratio was significantly higher (*p* < 0.05) in the BCB+ than in the BCB- oocytes (Table 3). Although the supplementation of BCB- oocytes with 100 ng/mL DKK1 increases the number of TE cells, the difference was deemed statistically non-significant vis-à-vis BCB- control oocytes.

**Table 3:** Total number of ICM and TE cells and ICM/TE ratio (expressed as the percentage) in blastocysts produced from BCB+, BCB- (control), and BCB- oocytes supplemented with 100 ng/mL Wnt inhibitor, DKK1.

Group	ICM	TE	ICM/TE
BCB+	37.8 ± 2.43^a^	139 ± 4.37^a^	27.09 ± 0.98^a^
BCB-	23.8 ± 1.74^b^	117.6 ± 2.51^b^	20.18 ± 1.19^b^
BCB- + 100 ng/mL DKK1	27.2 ± 1.72^b^	127.4 ± 2.42^b^	21.7 ± 1.57^b^

Values are presented as Mean ± SEM. Bars with different superscripts indicate a significant difference (*p* < 0.05).

### 3.7 Wnt pathway inhibition increases the expression of developmentally important genes

**Figure 5:**
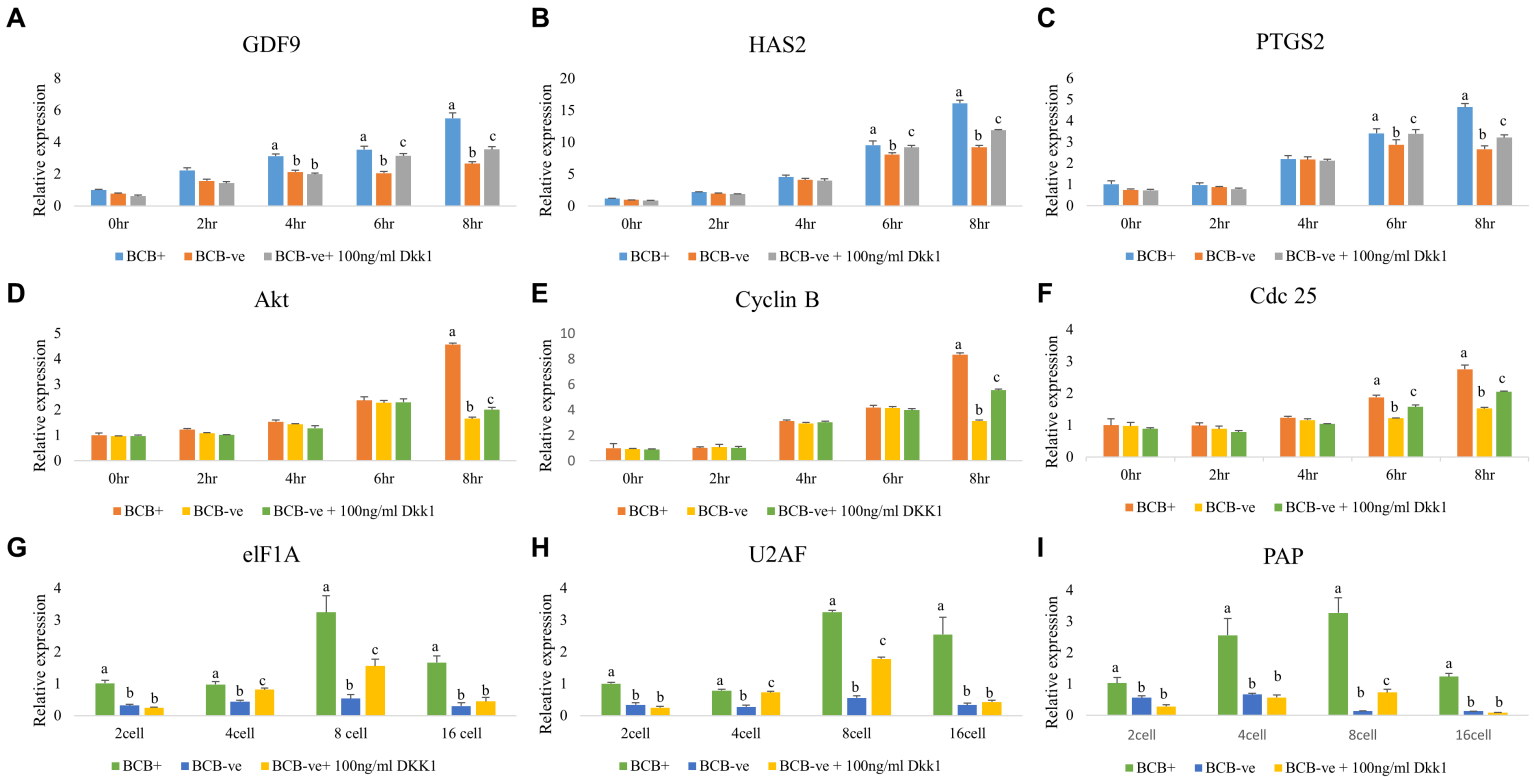
Expression pattern of developmentally important genes in poor-quality oocytes and embryos after Wnt pathway inhibition Real-time expression analysis of cumulus expansion **(A–C)**, GVBD **(D–F)**, and EGA **(G–I)** genes in BCB+, BCB- (control), and BCB- oocytes supplemented with 100 ng/mL Wnt inhibitor, DKK1 at various stages of IVM **(A–F)** and embryo development **(G–I)**. Values are presented as Mean ± SEM. Bars with different superscripts indicate a significant difference (*p* < 0.05).

The developmental ability of oocytes post-DKK1 supplementation was further assessed by monitoring the expression pattern of various genes involved in cumulus expansion, oocyte maturation and embryonic development (Figure 5). The expression of genes related to cumulus expansion elevated significantly (*p* < 0.05) at 6 h and 8 h of IVM upon DKK1 supplementation of BCB- oocytes (Figures 5A–C) while the expression of genes related to germinal vesicle breakdown in such oocytes increased (*p* < 0.05) at 8 h of IVM (Figures 5D–F). The expression of genes related to embryonic genome activation increased significantly (*p* < 0.05) at the 8-cell stage of embryonic development (Figures 5G–I).

## 4 Discussion

**Figure 6:**
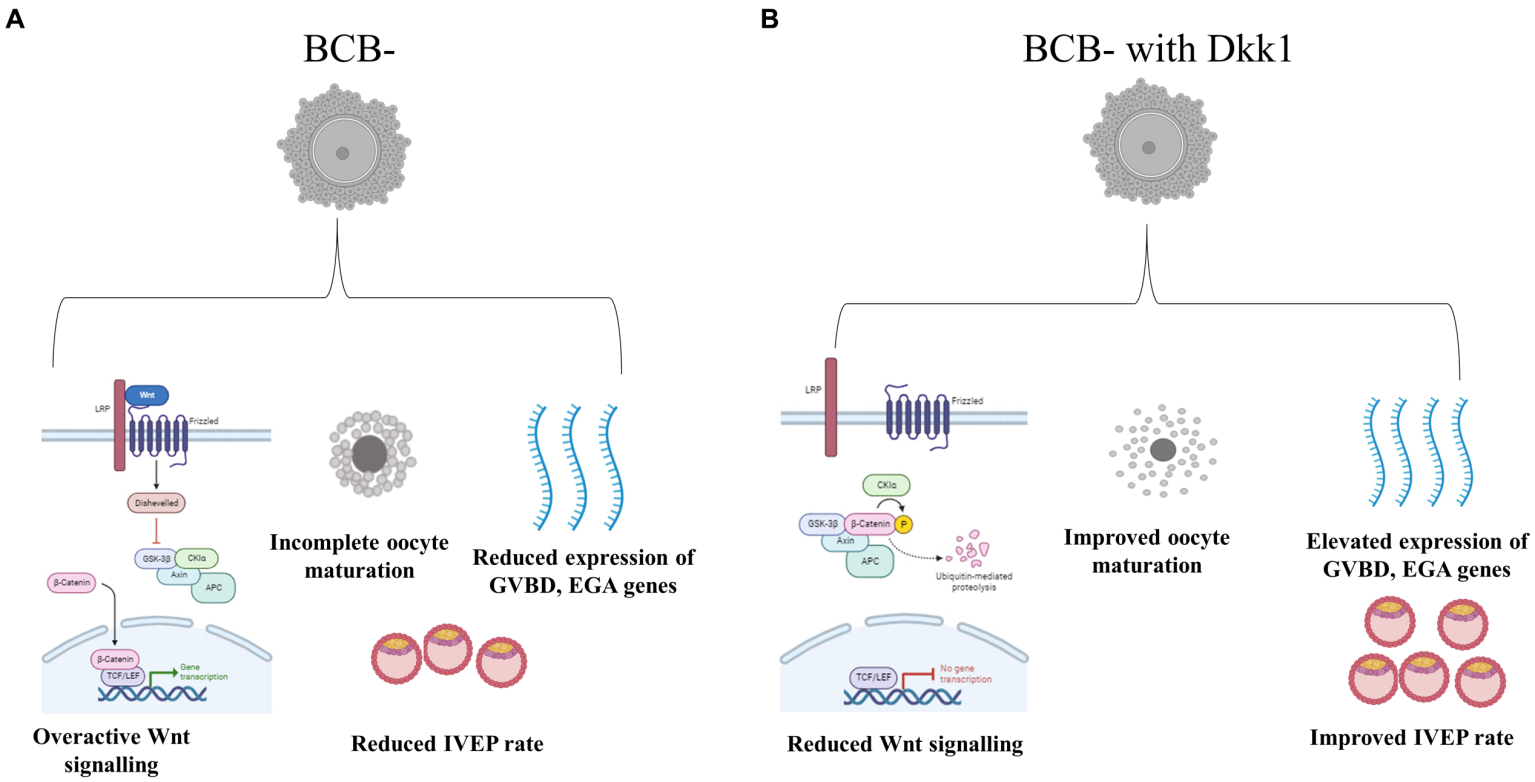
Schematic representation of the effect of Wnt pathway inhibition in BCB- oocytes **(A)**. Supplementation of BCB- oocytes with Wnt pathway antagonist DKK1 resulted in improved development competence of these poor-quality oocytes **(B)**.

The present study was undertaken to investigate whether the poor quality (BCB-) and good quality (BCB+) buffalo oocytes vary vis-à-vis the Wnt signaling pathway and whether the transient inhibition of this pathway at a pre-determined course of oocyte maturation improves the developmental competence of poor quality (BCB-) oocytes. We observed a differential expression of key Wnt genes in the BCB+ and BCB- oocytes, particularly at 8 h of IVM which was further substantiated by assessment of β-catenin protein abundance at this time point. The inhibition of the Wnt pathway in BCB- oocytes using a Wnt antagonist, DKK1 resulted in improved cumulus expansion characteristics, nuclear maturation rate and percentage of oocytes reaching the morula and blastocyst stages (Figure 6). Besides, the expression of developmentally important genes related to cumulus expansion, GVBD and EGA increased in the poor quality (BCB-) oocytes after DKK1 supplementation. The present data underpins the hypothesis that an overactive Wnt signaling pathway perturbs the development potential of growing oocytes and inhibition of this pathway during the initial course of oocyte maturation can improve the developmental ability of poor quality (BCB-) buffalo oocytes.

Although the BCB screening method provides a solid foundation for differentiating good-quality oocytes from poor-quality oocytes, limited evidence exists to support the variation in their developmental competence. The Wnt signaling pathway is among the most crucial cell–cell communication systems that play indispensable roles in numerous developmental processes, particularly during embryogenesis. It has been reported previously that the over-expression of these pathway genes in the *in vitro* matured oocytes compromises their quality vis-à-vis the *in vivo* matured oocytes (43). We observed the maximum difference in the Wnt pathway gene expression between the *in vitro* matured BCB+ and BCB- oocytes during the initial 8 h (Figures 1A–E). The initial stages of maturation are important since the most important milestone of oocyte development, i.e., GVBD is initiated during this time when the products of different genes required for embryogenesis start accumulating in the oocytes (56). We have previously reported that the GVBD in BCB- oocytes lags by 2–4 h in comparison to the BCB+ oocytes until ~10 h of IVM compromising the developmental ability of poor-quality (BCB-) buffalo oocytes. Nonetheless, the BCB- oocytes were observed to catch up their maturation rates (after 10 h) with that of BCB + oocytes (57). The highest difference in expression and abundance of Wnt pathway elements was observed at 8 h of IVM, therefore we hypothesize a similar synchronization of the Wnt pathway activity between the BCB+ and BCB- oocytes during the initial stages of IVM. It is well-established that each cell has an intrinsic ability to adjust its sensitivity to a signal. The same could be the case with the BCB- oocytes wherein a continuous Wnt signal may cause a swift and substantial increase in the number of endocytic vesicles causing temporary sequestration of receptor protein, e.g., FZD4 in the endosomes (58). However, further studies are warranted to validate this.

It has been reported that the Wnt signaling regulates the blastocyst development and inhibition of this pathway by DKK1 has been demonstrated to improve oocyte maturation (39, 59). Cumulus expansion can be considered as a morphological indicator of oocyte competence and BCB+ oocytes are known to have an optimum CEI which is associated with higher developmental competence (55). Our results agree with previous studies, which have also reported that the BCB+ oocytes have better cumulus expansion than BCB- oocytes (60). Nonetheless, we observed an improvement in the cumulus expansion characteristics of buffalo BCB- oocytes upon supplementation with 100 ng/mL DKK1 (Figure 2), as previously reported in pigs (43, 61). Cumulus expansion significantly influences the maturation of oocytes since the bidirectional crosstalk between the oocytes and the surrounding complex helps in the formation and deposition of an extracellular matrix between cumulus cells which aids in maturation (62). This crosstalk is facilitated by the presence of gap junctions. Cx43 is the major connexin protein that assists in the formation of gap junctions in cumulus cells and is implicated in the modulation of developmental competence (63, 64). A basal level of Wnt signaling, e.g., in BCB+ buffalo oocytes allows the cytoplasmic accumulation of Cx43, whereas an over-activated Wnt signaling will cause the nuclear export of Cx43 and its association with β-catenin destruction complex hence disrupting the gap junctions (65). However, in the BCB- oocytes supplemented with DKK1, the cytoplasmic Cx43 and β-catenin levels are reportedly restored which assists in the formation of gap junctions leading to enhanced cumulus expansion. The supplementation of DKK1 also improved the mean percentage of fertilized oocytes reaching the morula and blastocyst stages in BCB- oocytes (Table 1). Nonetheless, there was no significant difference in the percentage of oocytes that were cleaved or reached the 8–16 cell stage between the BCB-control and BCB- oocytes supplemented with DKK1. It has been reported that DKK1 is less effective in situations where the Wnt activity is essential for cell survival (39, 40). Besides, it has been established that the continuous activation of the Wnt signaling pathway in oocytes does not affect its functions, e.g., folliculogenesis, however, negatively affects blastocyst implantation and embryonic and fetal development (66, 67). Our results are also supported by similar studies which have reported that an activated Wnt signaling led to reduced hatching of blastocysts and a lesser number of nuclei per blastocyst (68). We observed a significant reduction in the level of β-catenin (and Dvl1) gene expression in BCB- oocytes upon DKK1 supplementation. It has been reported that embryos fail to develop beyond the 2-cell stage due to atypical expression of β-catenin (69). In the case of BCB- oocytes, an overactive Wnt signal may cause early translocation of β-catenin inside the nucleus which can cause transcriptional activation of genes like Tap63 which are implicated in determining the fate in oocytes of primordial follicles (70, 71). However, this aspect needs further investigation to establish the effect of DKK1 supplementation on BCB- oocytes.

DKK1 supplementation improved the percentage of BCB- oocytes that matured to the metaphase II stage (Table 2). Our results corroborate the findings in pigs wherein DKK1 supplementation improved the *in vitro* nuclear maturation rate of oocytes (43). It is highly likely that DKK1 supplementation of BCB- oocytes improves the nuclear maturation of these oocytes by accelerating the downstream fatty acid oxidation in poor-quality (BCB-) COCs (72), nonetheless, further studies are warranted to establish this effect. It has been reported that exposure of bovine embryos to DKK1 results in elevated differentiation of embryonic cells toward the trophectoderm and hypoblast lineages, however, it does not promote cell proliferation in early embryos (40). No effect on blastocyst health (ICM/TE ratio) of BCB- oocytes upon DKK1 supplementation was observed in this study. Our results from differential staining along with the results of IVEP experiments indicate that although DKK1 is required for blastocyst development, it does not regulate ICM proliferation (40, 73).

Based on the CEI score and increased expression of GDF9, HAS2, and PTGS2 upon Wnt inhibition, we inferred that DKK1 helps the poor-quality (BCB-) buffalo oocytes to gain competence. Our data is in line with the previous findings that reported the maximum expression of these genes during the mid-maturation phase (52). However, to understand the reason behind the increased oocyte maturation of BCB- oocytes supplemented with DKK1, we considered the two other important milestones of embryonic development, i.e., GVBD and EGA (Figure 5). The inhibition of the Wnt pathway by DKK1 assists BCB- oocytes to synchronize their timing of GVBD with that of BCB+ oocytes (57, 67). A cell surface receptor for DKK1, CKAP4 (Cytoskeleton associated protein 4), could be implicated in this process. It is now known that upon binding with the DKK1, CKAP4 recruits PI3K to its cytosolic domain which promotes Akt signaling thereby triggering the event of GVBD in BCB- oocytes (74). Besides, the genes elF1A, PAP, and U2AF are considered authentic indicators of EGA and are involved in the DNA and RNA metabolism in oocytes (75). These genes exhibited minimal expression in BCB- (control) oocytes, which increased upon DKK1 supplementation during the 4–8 cell stage which is the time when major embryonic genome activation takes place in buffaloes (75). During EGA, the endogenous molecules responsible for inhibiting canonical Wnt signaling are already present. Additionally, the calcium fluctuations at the time of fertilization also inhibit the canonical Wnt signaling (76). The combined effect of endogenous molecules along with the exogenous supplementation of DKK1 might be responsible for overcoming the negative effects of hyperactive Wnt signaling and hence helping the BCB- oocytes to achieve the milestone of MET earlier than the BCB- control group (57, 67).

The presented results confirm the presence of an overactive Wnt signaling pathway in BCB- oocytes and strengthen the hypothesis that hyperactive Wnt signaling perturbs the development potential of poor-quality oocytes. Transient inhibition of Wnt signaling using DKK1 during the initial course of oocyte maturation can prove to be a feasible strategy for attaining better embryonic development in poor-quality buffalo oocytes in the IVEP regimen.

## Data availability statement

The original contributions presented in the study are included in the article/Supplementary material, further inquiries can be directed to the corresponding author.

## Ethics statement

Ethical approval was not required for the study involving animals in accordance with the local legislation and institutional requirements because The study was performed on samples procured from an abattoir.

## Author contributions

KA: Conceptualization, Data curation, Investigation, Methodology, Validation, Visualization, Writing – original draft, Writing – review & editing. VB: Investigation, Methodology, Visualization, Writing – original draft, Writing – review & editing. RK: Conceptualization, Project administration, Resources, Supervision, Writing – review & editing. TD: Conceptualization, Data curation, Funding acquisition, Investigation, Project administration, Resources, Supervision, Writing – review & editing.

## Supplementary material

Supplementary Figure 1 Immunostaining of differentially screened BCB oocytes with anti-β-catenin antibody. **(A)** Control oocytes without any primary antibody. **(B)** Abundance of β-catenin in BCB- oocytes. **(C)** Abundance of β-catenin in BCB+ oocytes at various hours of IVM.

Supplementary Figure 2 Different scales of cumulus expansion. **(A)**, CEI=0 (No expansion); **(B)**, CEI=1 (Outer layers expanded); **(C)**, CEI=2 (50% of the layers expanded); **(D)**, CEI=3 (All layers expanded).
