# Supplementary material for: Transient suppression of Wnt signaling in poor-quality buffalo oocytes improves their developmental competence
Source: Front Vet Sci. 2024 Jan 11;10:1324647. doi: 10.3389/fvets.2023.1324647 (PMC10808588; doi:10.3389/fvets.2023.1324647)
Supplement: Supplementary file 1 [file Table_1.docx]

**Supplementary Table 1- List of primers used for RT-qPCR expression analysis**

| DVL1 Forward | 5’ TTTCAGCAGCATCACGGACT 3’ |
| --- | --- |
| DVL1 Reverse | 5’ AGTGGTGCCTCTCCATGTTG 3’ |
| FZD4 Forward | 5’ CACCATTGACTTCCAGCACG 3’ |
| FZD4 Reverse | 5’ ATAAACTGGCAGCTCCTCGC 3’ |
| WNT7A Forward | 5’ ACAAGTACAACGAGGCGGTC 3’ |
| WNT7A Reverse | 5’ GGACAGCGGCTTCTTGATCT 3’ |
| WNT3A Forward | 5’ TCCTCTACGGCCTGAAGCAAG 3’ |
| WNT3A Reverse | 5’ GAGTACTGGAGCCCAACAGC 3’ |
| β Catenin Forward | 5’ GCTTCCTTAAAGGCGAGGGT 3’ |
| β Catenin Reverse | 5’ AATCATGGCATGGTCCAGCA 3’ |
| AKT Forward | 5’ CTTCAAGCCTCAGGTCACATC 3’ |
| AKT Reverse | 5’ CATGCTGTCGTCTTGGTCAG 3’ |
| CDC25B Forward | 5’ AGAAACGGTGGTGGCTCT 3’ |
| CDC25B Reverse | 5’ AGGGGTATCTGCAGTCAACA 3’ |
| Cyclin B Forward | 5’ CATTTCCTTCGGAGAGCATC 3’ |
| Cyclin B Reverse | 5′-AGAAGGAGGAAAGTGCACCA-3′ |
| GDF9 Forward | 5’ TCAGCACAAGCAAGCTCCT 3’ |
| GDF9 Reverse | 5’ GGGAAGGGAAAAGAAATGGA 3’ |
| RPS18 Forward | 5’ GAAAATTGCCTTTGCCATCACTGC 3’ |
| RPS18 Reverse | 5’ GATCACACGTTCCACCTCATCCTC 3’ |
| eIF1A Forward | 5' CTCCCAAGGGCTGAGAAAG 3' |
| eIF1A Reverse | 5' TCACTCTCCTCCTCGCTCTC 3‘ |
| U2AF Forward | 5' GATGTCGAGATGCAGGAACA 3’ |
| U2AF Reverse | 5' TCTTCTTCA CGGCGAAACTT 3' |
| PAP Forward | 5’ GGATGGTCATGGTTGAGGAG 3’ |
| PAP Reverse | 5’ GGCGTTGTTTTTCAGTTGGT 3’ |
