# Supplementary figures and images for: Transient suppression of Wnt signaling in poor-quality buffalo oocytes improves their developmental competence
Source: Front Vet Sci. 2024 Jan 11;10:1324647. doi: 10.3389/fvets.2023.1324647 (PMC10808588; doi:10.3389/fvets.2023.1324647)

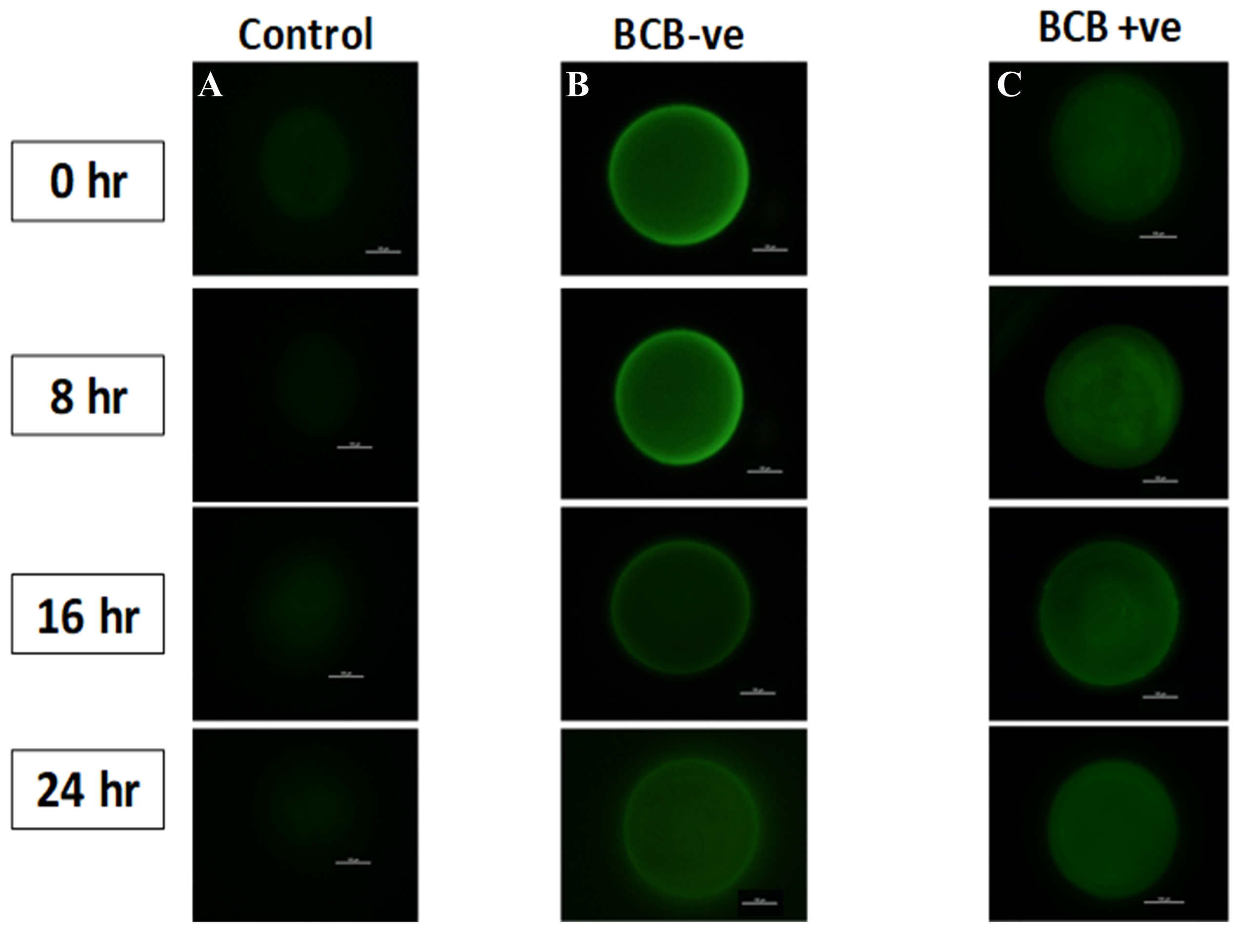

Supplement: Supplementary Figure 1 — Immunostaining of differentially screened BCB oocytes with anti-β-catenin antibody. (A) Control oocytes without any primary antibody. (B) Abundance of β-catenin in BCB- oocytes. (C) Abundance of β-catenin in BCB+ oocytes at various hours of IVM. [file Image_1.tif]

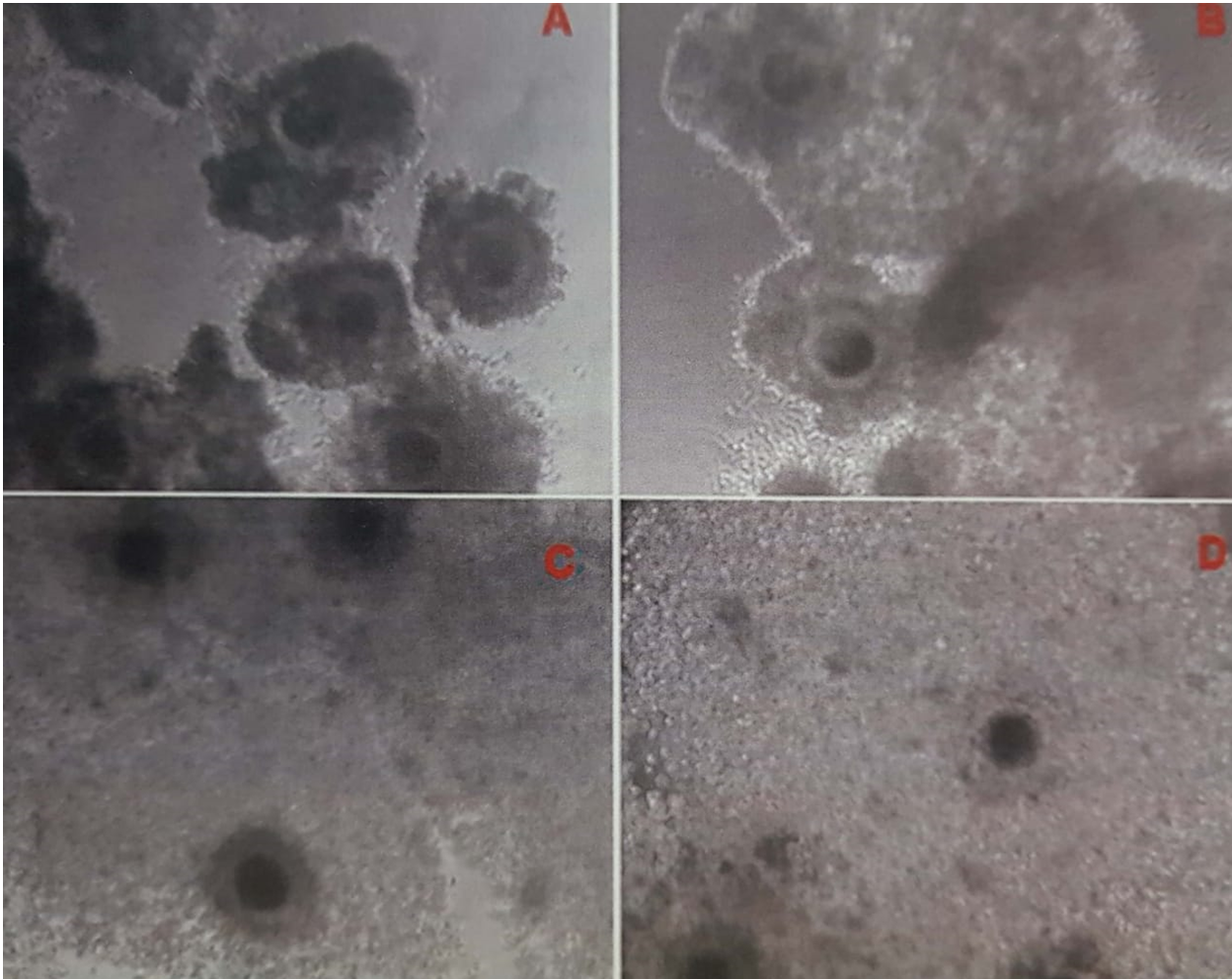

Supplement: Supplementary Figure 2 — Different scales of cumulus expansion. (A), CEI=0 (No expansion); (B), CEI=1 (Outer layers expanded); (C), CEI=2 (50% of the layers expanded); (D), CEI=3 (All layers expanded). [file Image_2.tif]
